# Bayes rules for optimally using Bayesian hierarchical regression models in provider profiling to identify high-mortality hospitals

**DOI:** 10.1186/1471-2288-8-30

**Published:** 2008-05-12

**Authors:** Peter C Austin

**Affiliations:** 1 Institute for Clinical Evaluative Sciences, Toronto, Ontario; 2 Department of Public Health Sciences, University of Toronto, Toronto, Ontario; 3 Department of Health Policy, Management, and Evaluation, University of Toronto, Canada

## Abstract

**Background:**

There is a growing trend towards the production of "hospital report-cards" in which hospitals with higher than acceptable mortality rates are identified. Several commentators have advocated for the use of Bayesian hierarchical models in provider profiling. Several researchers have shown that some degree of misclassification will result when hospital report cards are produced. The impact of misclassifying hospital performance can be quantified using different loss functions.

**Methods:**

We propose several families of loss functions for hospital report cards and then develop Bayes rules for these families of loss functions. The resultant Bayes rules minimize the expected loss arising from misclassifying hospital performance. We develop Bayes rules for generalized 1-0 loss functions, generalized absolute error loss functions, and for generalized squared error loss functions. We then illustrate the application of these decision rules on a sample of 19,757 patients hospitalized with an acute myocardial infarction at 163 hospitals.

**Results:**

We found that the number of hospitals classified as having higher than acceptable mortality is affected by the relative penalty assigned to false negatives compared to false positives. However, the choice of loss function family had a lesser impact upon which hospitals were identified as having higher than acceptable mortality.

**Conclusion:**

The design of hospital report cards can be placed in a decision-theoretic framework. This allows researchers to minimize costs arising from the misclassification of hospitals. The choice of loss function can affect the classification of a small number of hospitals.

## Background

Public reporting of comparative health care performance by hospitals is part of current public policy in a range of jurisdictions. Several jurisdictions have released public report cards comparing hospital or physician-specific outcomes. California [1], Pennsylvania [2], Scotland [3], and Ontario, Canada [4] have released hospital-specific reports for mortality following admission for acute myocardial infarction (AMI). New Jersey [5], New York [6], Pennsylvania [7], and Massachusetts [8] have published hospital and surgeon-specific mortality rates following coronary artery bypass graft (CABG) surgery, while Ontario has published hospital-specific mortality rates [9].

It has been suggested that there are two main goals behind public reporting [10]. The first is providing information that can guide purchasing decisions by individual consumers or group purchasers such as employers or health maintenance organizations (HMOs). The alternative rationale for public performance reporting is to identify hospitals that require investment in quality improvement initiatives. Implicit in this theoretical framework for hospital report cards is the ability to identify hospitals from which one does not want to seek care, or that require investment in quality improvement initiatives. Furthermore, it implies that we are interested not just in a point estimate of a hospital's mortality rate or relative ranking, but in making a decision about whether to seek care from that hospital or about whether to invest in quality improvement initiatives at that hospital. Normand and Shahian discuss statistical and clinical issues related to provider profiling [11].

Several authors have demonstrated that even if perfect risk-adjustment was possible, random error will result in some hospitals being misclassified [12-14]. Different participants in the health care arena will place different values or costs on different types of misclassifications and on the degree of misclassification. There are two types of misclassifications: false positives (hospitals that truly had acceptably mortality but that were classified as having unacceptably high mortality) and false negatives (hospitals that truly had unacceptably high mortality but were classified as having acceptable mortality). A health care consumer might place higher value on information that minimizes false negatives – they want to avoid purchasing or receiving care from a hospital with unacceptably high mortality. On the other hand, hospitals might put a higher value on information that minimizes false positives – they want to avoid losing business if they are not low performers. The same argument could be made regarding targeting hospitals for quality improvement – false negatives would lead to lost opportunities to invest in quality improvement and false positives would lead to unneeded investment in quality improvement.

Several investigators have suggested the use of Bayesian methods in provider profiling [15-17]. Spiegelhalter et al. used Bayesian methods to examine hospital-specific mortality rates following paediatric cardiac surgery [18]. Thomas et al. used an empirical Bayes model to estimate hospital-level mortality rates for Medicare patients in the United States [19]. Investigators have argued for these methods since they allow profiling to be guided by medical rather than statistical standards, allow hospitals with small caseloads to be included, eliminate regression to the mean bias, and allow the probability of acceptable provider performance to be calculated. Recently Austin examined the reliability and validity of four different Bayesian measures of hospital performance using Monte Carlo simulation methods [20]. Furthermore, the agreement between four different Bayesian methods was examined empirically [21].

Normand et al. proposed a method for provider profiling based upon posterior tail probabilities [16]. Recently, Austin and Brunner used Monte Carlo simulations to assess the accuracy of posterior tail probabilities derived from Bayesian hierarchical regression models for identifying hospitals with higher than acceptable mortality [13]. In doing so, they demonstrated that the use of posterior tail probabilities was the Bayes Rule associated with generalized 1-0 loss functions. However, beyond this initial result, Bayes rules for more complex loss functions have not been derived for hospitals report cards. Furthermore, the impact of assuming different loss functions on the identification of hospitals with higher than acceptable mortality when Bayesian methods are employed has not been explored in the literature.

Accordingly, the objective of the current manuscript is two-fold. First, to develop Bayes rules for several families of loss functions for hospitals report cards when Bayesian hierarchical models are used. Second, to demonstrate the impact of assuming different loss functions on the number of hospitals identified as having unacceptably high mortality.

### Statistical model for classifying hospitals as mortality outliers

In this study, we used methods for provider profiling based on a Bayesian hierarchical regression model. Let p_ij _denote the probability of mortality for the i^th ^patient treated at the j^th ^hospital. Furthermore, let X_ij _denote the illness severity of this patient, which was assumed to be centered around the cohort average. Then the following model describes the relationship between mortality and illness severity:

logit(p_ij_) = β_0j _+ β_1j_X_ij_,

In this model, it is assumed that $\left(\begin{array}{l} \beta_{0j}\\ \beta_{1j} \end{array}\right)$, the hospital-specific vector of regression coefficients followed a multivariate normal distribution: $\left(\begin{array}{l} \beta_{0j}\\ \beta_{1j} \end{array}\right)\sim MVN\left(\left(\begin{array}{l} \beta_{0}\\ \beta_{1} \end{array}\right),\left(\begin{array}{cc} \sigma_{1}^{2} & \sigma_{12}\\ \sigma_{12} & \sigma_{2}^{2} \end{array}\right)\right)$. Here, β_0j _denotes the hospital-specific log-odds of death for an average patient, β_1j _denotes the hospital-specific regression slope relating illness severity to the log-odds of death, and $\left(\begin{array}{cc} \sigma_{1}^{2} & \sigma_{12}\\ \sigma_{12} & \sigma_{2}^{2} \end{array}\right)$ denotes the variance-covariance matrix for the hospital-specific random effects. β_0 _denotes the mean hospital-specific log-odds of mortality for a patient, with average disease severity, in the population of hospitals. Finally, β_1 _denotes the mean hospital-specific slope relating illness severity and the log-odds of mortality, in the population of hospitals.

### Bayes Rules for provider profiling

In this section, we develop Bayes rules for specific cost functions. The resultant Bayes rules minimize the posterior expected cost due to incorrectly classifying hospital performance.

#### Notation

Let *π *(*θ*, *X*) denote the prior distribution of the parameter θ, which denotes an individual hospital's random intercept. Let *β*_*thresh *_denote the threshold for defining acceptable quality of care. Then *θ*_0 _= {*θ *|*θ *<*β*_*thresh*_} denotes the space of random effects that define hospitals with acceptable mortality, and $\theta_{0}^{c}=\{\theta |\theta >\beta_{thresh}\}$ denotes the space of random effects that define hospitals with unacceptably high mortality. Our definition of profiling is based on *θ *= *β*_0*j*_, the hospital-specific random intercept, which denotes the hospital-specific log-odds of death for an average patient in the population of patients. We exclude the random slopes, *β*_1*j*_, from the definition of mortality outliers due to a lack of consensus on how to identify performance outliers using both random intercepts and random slopes. Furthermore, the intercept has a readily understandable interpretation that is related to mortality. As discussed by Gajewski et al., the choice of *β*_*thresh *_is primarily a clinical, rather than a statistical, decision [22]. For instance, the choice of *β*_*thresh *_can be informed by expert opinion as to what constitutes acceptable mortality for an average AMI patient.

Let the decision rule *d *be 1 if one decides that the hospital has poor performance and 0 if one decides that the hospital has acceptable performance. Then, we define a general loss function

*L*(*θ*, *d*) = *f*_1_(*θ*)*dI*(*θ *<*β*_*thresh*_) + *f*_2_(*θ*)(1-*d*)I(*θ *> *β*_*thresh*_)

Here *dI*(*θ *<*β*_*thresh*_) denotes a false positive: classifying a hospital as having higher than acceptable mortality when in reality it has acceptable mortality. *f*_1_(*θ*) is the cost occurred for false positives. Similarly, (1-*d*)*I*(*θ *> *β*_*thresh*_) denotes a false-negative: classifying a hospital as having acceptable performance when in reality it has poor performance. *f*_2_(*θ*) is the cost occurred for false negatives. In the above, *I *denotes the indicator function that takes the value 1 if the condition is true and 0 otherwise. Let H_0 _denote the null hypothesis that the hospital has an acceptable mortality rate. In the following sections, we shall derive Bayes rules for different families of cost functions.

#### Identifying Bayes Rules

Let *R*(*x*, *d *= 0) denote the cost associated with deciding that the hospital has acceptable mortality, while *R*(*x*, *d *= 1) denotes the cost associated with deciding that the hospital has unacceptably high mortality. Then

*R*(*x, d *= 0) = ∫ *f*_2_(*θ*)*I*(*θ *> *β*_*thresh*_)*π *(*θ *|*x*)*dθ*

and

*R*(*x, d *= 1) = ∫ *f*_1_(*θ*)*I*(*θ *<*β*_*thresh*_)*π *(*θ *|*x*)*dθ*

In each case, the integral is over the space of the random effects. Then, to minimize cost, we reject H_0 _only if *R*(*x, d *= 1) <*R*(*x*, *d *= 0). A decision is said to be optimal if it minimizes the posterior expected loss under a specified loss function [23,24].

#### Cost functions

We consider three different families of loss functions. The first is the family of generalized 1-0 loss functions. The second is the family based on absolute error loss functions, while the third is based on squared error loss. Within each family, we consider symmetric and asymmetric loss functions. Among the set of asymmetric loss functions, we will consider loss functions that penalize false positives more heavily than false negatives, and loss functions that penalize false negatives more heavily than false positives. Symmetric loss functions penalize false positives and false negatives equally.

#### Generalized 1-0 loss functions

In this section, we assume a generalized 1-0 loss function. Let H_0 _denote the null hypothesis that the hospital delivers acceptable quality care, c_I _be the penalty associated with a type I error, and c_II _be the penalty associated with a type II error. Then the loss function for generalized 1-0 loss is

*c*_*I *_*dI*(*θ *<*β*_*thresh*_) + *c*_*II *_(1-*d*)*I*(*θ *> *β*_*thresh*_)

In an earlier paper, Austin and Brunner derived Bayes rules for generalized 1-0 loss functions [13]. It was shown that to minimize risk, we reject H_0 _only if

$$P(\theta >\beta_{thresh}|X)>\frac{c_{I}}{c_{I}+c_{II}}=P_{0}$$

Here P_0 _is the optimum posterior tail probability. Thus, the use of a generalized 1-0 loss function results in a Bayes Rule based on posterior tail probabilities. For instance, if *c*_*I *_= *c*_*II *_= 1, then *P*_0 _= 0.5. Thus, the use of a 1-0 loss function results in a Bayes Rule that says that a hospital should be classified as having unacceptably high mortality if the posterior tail probability that the hospital-specific random intercept exceeds the threshold of acceptable care with a probability of at least 0.5. The reader is referred to the original article for greater details. One should observe that this result is independent of the distribution of the random effects and of the prior distributions.

We re-express the generalized 1-0 loss function as follows for consistency with the subsequent sections:

*dI*(*θ *<*β*_*thresh*_) + *k*(1-*d*)*I*(*θ *> *β*_*thresh*_)

Here $k=\frac{c_{II}}{c_{I}}$ denotes the relative loss incurred by false negatives compared to that incurred by false positives. We now derive the Bayes rules as an expectation so as to maintain consistency with subsequent sections.

$$\begin{array}{c} R(x,d=0)=\int_{-\infty }^{\infty }kI(\theta >\beta_{thresh})\pi (\theta |x)d\theta \\ =\int_{\beta_{thresh}}^{\infty }k\pi (\theta |x)d\theta \end{array}$$

Similarly,

$$\begin{array}{lll} R(x,d=1) & = & \int_{-\infty }^{\beta_{thresh}}\pi (\theta |x)d\theta \\ & = & \text{1 }-\int_{\beta_{thresh}}^{\infty }\pi (\theta |x)d\theta \end{array}$$

Then,

$$\begin{array}{l} R(x,d=1)<R(x,d=0)\Leftrightarrow \\ 1-\int_{\beta_{thresh}}^{\infty }\pi (\theta |x)d\theta <k\int_{\beta_{thresh}}^{\infty }\pi (\theta |x)d\theta \Leftrightarrow \\ (k+1)\int_{\beta_{thresh}}^{\infty }\pi (\theta |x)d\theta >1\Leftrightarrow \\ \int_{\beta_{thresh}}^{\infty }\pi (\theta |x)d\theta >\frac{1}{k+1}\Leftrightarrow \\ \int_{\beta_{thresh}}^{\infty }I(\theta >\beta_{thresh})\pi (\theta |x)d\theta -\frac{1}{k+1}>0\Leftrightarrow \\ \int_{-\infty }^{\infty }I(\theta >\beta_{thresh})\pi (\theta |x)d\theta -\frac{1}{k+1}>0\Leftrightarrow \\ E\left[\begin{array}{c} I(\theta >\beta_{thresh}) \end{array}-\frac{1}{k+1}\right]>0 \end{array}$$

We have now expressed the Bayes Rule for a generalized 1-0 loss function as an expectation of a function of the model parameters and the cost penalty for false negatives.

These Bayes rules can be evaluated using Markov Chain Monte Carlo (MCMC) methods. To do so, for each hospital, one evaluates the expression $I(\theta >\beta_{thresh})-\frac{1}{k+1}$ at each iteration of the MCMC simulation. One then determines the expectation or mean of this quantity over all the iterations of the MCMC simulations. If the mean of this quantity is larger than zero, the hospital is classified as a high-mortality outlier. If the mean of the quantity is less than zero, the hospital is classified as a low-mortality outlier.

Generalized absolute error loss functions

Let H_0 _denote the null hypothesis that the hospital delivers acceptable quality care. Let *β*_*thresh *_denote the threshold that denotes acceptable quality of care, and let *θ *denote a given hospital's log-odds of death for an average patient. We define d = 1 to be the decision that a hospital has unacceptably high mortality, while d = 0 is the decision that a hospital has acceptable mortality. We then define the following loss function:

|*θ *- *β*_*thresh *_|*dI*(*θ *<*β*_*thresh*_) + *k*|*θ *- *β*_*thresh *_| (1-*d*)*I*(*θ *> *β*_*thresh*_)

In this loss function, the penalty for misclassification is a linear function of the absolute difference between the threshold for acceptable mortality and the hospital's actual random effect. False negatives are penalized with a penalty of *k *| *θ *- *β*_*thresh *_|, whereas false positives are penalized with a penalty of | *θ *- *β*_*thresh *_|. Thus, this loss function allows for the possibility of asymmetry. We describe it as a generalized absolute error loss function. Then we have that

$$\begin{array}{ccc} R(x,d=0) & = & \int_{\theta_{0}^{c}}k(\theta -\beta_{thresh})\pi (\theta |x)d\theta \\ & = & \int_{\beta_{thresh}}^{\infty }k(\theta -\beta_{thresh})\pi (\theta |x)d\theta \end{array}$$

Similarly,

$$R(x,d=1)=\int_{-\infty }^{\beta_{thresh}}(\beta_{thresh}-\theta )\pi (\theta |x)d\theta$$

Then,

$$\begin{array}{l} R(x,d=1)<R(x,d=0)\Leftrightarrow \\ \int_{-\infty }^{\beta_{thresh}}(\beta_{thresh}-\theta )\pi (\theta |x)d\theta <k\int_{\beta_{thresh}}^{\infty }(\theta -\beta_{thresh})\pi (\theta |x)d\theta \Leftrightarrow \\ -\int_{-\infty }^{\beta_{thresh}}(\beta_{thresh}-\theta )\pi (\theta |x)d\theta +\;k\int_{\beta_{thresh}}^{\infty }(\theta -\beta_{thresh})\pi (\theta |x)d\theta >0\Leftrightarrow \\ \int_{-\infty }^{\beta_{thresh}}(\theta -\beta_{thresh})\pi (\theta |x)d\theta +\;k\int_{\beta_{thresh}}^{\infty }(\theta -\beta_{thresh})\pi (\theta |x)d\theta >0\Leftrightarrow \\ \int_{-\infty }^{\infty }(\theta -\beta_{thresh})I(\theta <\beta_{thresh})\pi (\theta |x)d\theta +\;k\int_{-\infty }^{\infty }(\theta -\beta_{thresh})I(\theta >\beta_{thresh})\pi (\theta |x)d\theta >0\Leftrightarrow \\ E[(\theta -\beta_{thresh})I(\beta_{thresh}-\theta )]+kE[(\theta -\beta_{thresh})I(\theta -\beta_{thresh})]>0 \end{array}$$

The final inequality above is the Bayes Rule for a generalized absolute error loss function. As with the generalized 1-0 loss function, the Bayes Rule has been expressed as an expectation of the model parameters, the threshold for acceptable mortality, and the loss parameter k.

In the special case in which false positives and false negatives are penalized equally, which corresponds to allowing k to be 1 in the above formulas, the Bayes Rule reduces to:

*E *[(*θ *- *β*_*thresh*_)*I*(*β*_*thresh *_- *θ*)] + *E *[(*θ *- *β*_*thresh*_)*I*(*θ *- *β*_*thresh*_)] > 0 ⇔

*E *[(*θ *- *β*_*thresh*_)(*I*(*β*_*thresh *_- *θ*)] + *I*(*θ *- *β*_*thresh*_))] > 0 ⇔

*E *[*θ *- *β*_*thresh*_] > 0

Therefore, in the case of symmetric linear loss, the resultant Bayes rule is that a hospital is classified as a high-mortality outlier if and only if the posterior mean of the hospital random intercept parameter is greater than the chosen threshold.

As with generalized 1-0 loss, these Bayes rules can be evaluated using Markov Chain Monte Carlo (MCMC) methods. To do so, for each hospital, one evaluates the expression (*θ *- *β*_*thresh*_)*I*(*β*_*thresh *_- *θ*)] + *kE *[(*θ *- *β*_*thresh*_)*I*(*θ *- *β*_*thresh*_)] at each iteration of the MCMC simulation. One then determines the expectation or mean of this quantity over all the iterations of the MCMC simulations. If the mean of this quantity is larger than zero, the hospital is classified as a high-mortality outlier. If the mean of the quantity is less than zero, the hospital is classified as a low-mortality outlier.

Generalized squared error loss functions

Let *β*_*thresh *_and *θ *be as above. We define the following loss function:

(*θ *- *β*_*thresh*_)^2^*dI*(*θ *<*β*_*thresh*_) + *k*(*θ *- *β*_*thresh*_)^2^(1 - *d*)*I*(*θ *> *β*_*thresh*_)

In this loss function, the penalty for misclassification is a quadratic function of the difference between the threshold for acceptable mortality and the hospital's actual random effect. False negatives are penalized with a penalty of *k*(*θ *- *β*_*thresh*_)^2^, whereas false positives are penalized with a penalty of (*θ *- *β*_*thresh*_)^2^. Thus, this loss functions allows for the possibility of asymmetry. Then

$$\begin{array}{lll} R(x,d=0) & = & \int_{\theta }L(\theta ,a_{0})\pi (\theta |x)d\theta \\ & = & \int_{\theta }k{\left(\theta -\beta_{thresh}\right)}^{2}\pi (\theta |x)d\theta \\ & = & \int_{\beta_{thresh}}^{\infty }k{\left(\theta -\beta_{thresh}\right)}^{2}\pi (\theta |x)d\theta \end{array}$$

Similarly,

$$\begin{array}{l} R(x,d=1)=\int_{-\infty }^{\beta_{thresh}}L(\theta ,a_{1})\pi (\theta |x)d\theta \\ R(x,d=1)=\int_{-\infty_{}}^{\beta_{thresh}}{\left(\beta_{thresh}-\theta \right)}^{2}\pi (\theta |x)d\theta \end{array}$$

Then,

$$\begin{array}{l} R(x,d=1)<r(x,d=0)\Leftrightarrow \\ \int_{-\infty }^{\beta_{thresh}}{\left(\beta_{thresh}-\theta \right)}^{2}\pi (\theta |x)d\theta <\int_{\beta_{thresh}}^{\infty }k{\left(\theta -\beta_{thresh}\right)}^{2}\pi (\theta |x)d\theta \Leftrightarrow \\ -\int_{-\infty }^{\beta_{thresh}}{\left(\beta_{thresh}-\theta \right)}^{2}\pi (\theta |x)d\theta +\;\int_{\beta_{thresh}}^{\infty }k{\left(\theta -\beta_{thresh}\right)}^{2}\pi (\theta |x)d\theta >0\Leftrightarrow \\ -\int_{-\infty }^{\infty }{\left(\theta -\beta_{thresh}\right)}^{2}I(\theta <\beta_{thresh})\pi (\theta |x)d\theta +\;\int_{-\infty }^{\infty }k{\left(\theta -\beta_{thresh}\right)}^{2}I(\theta >\beta_{thresh})\pi (\theta |x)d\theta >0\Leftrightarrow \\ -E[{\left(\theta -\beta_{thresh}\right)}^{2}I(\theta <\beta_{thresh})]+E[k{\left(\theta -\beta_{thresh}\right)}^{2}I(\theta >\beta_{thresh})]>0\Leftrightarrow \\ E[{\left(\theta -\beta_{thresh}\right)}^{2}(kI(\theta >\beta_{thresh})-I(\theta <\beta_{thresh}))]>0 \end{array}$$

The final inequality above is the Bayes Rule for a generalized squared error loss function. In the special case in which false positives and false negatives are penalized equally, which corresponds to allowing k to be 1 in the above formulas, the Bayes Rule reduces to:

*E *[(*θ *- *β*_*thresh*_)^2^(*I*(*θ *> *β*_*thresh*_)] - *I*(*θ *<*β*_*thresh*_))] > 0

As with generalized 1-0 loss and generalized absolute error loss, this decision rule can be computed using Markov Chain Monte Carlo (MCMC) methods.

### Case study

In this section we apply the Bayes rules derived above to a specific dataset to examine the impact of assuming different loss functions on the identification of hospitals with unacceptably high mortality.

#### Data sources

We used data on all 19,757 patients discharged from hospital with a most responsible diagnosis of acute myocardial infarction (AMI) between April 1, 2000 and March 31, 2001 from the 163 acute care hospitals in Ontario, Canada that treated at least 1 AMI patient during the 12 month period. Creation of this dataset is described in detail elsewhere [4,25].

Adjustments for differences in case-mix were done using the Ontario AMI mortality prediction rule for 30-day mortality, whose derivation and validation are described elsewhere [26]. The variables comprising the prediction rule consisted of age, gender, cardiac severity (e.g., congestive heart failure, cardiogenic shock, arrhythmia, and pulmonary edema), and comorbid status (e.g., diabetes mellitus with complications, stroke, acute and chronic renal disease, and malignancy), as derived from the ICD-9 codes present in the 15 secondary diagnostic fields of the hospitalization database.

An illness severity score was derived as the predicted probability of 30-day mortality using age, gender and the 9 risk factors and comorbidities comprising the Ontario AMI mortality prediction rule. This method of constructing an illness severity score has been used elsewhere [27,28]. The illness severity scores were then standardized so as to have mean 0 and variance 1. Thus, a patient with average disease severity had a disease severity score of 0.

#### Cost functions

In this case study we consider 9 different loss functions: three generalized 1-0 loss functions, 3 generalized absolute error loss functions, and 3 generalized squared error loss functions. We used values of k of 1, 2, and 0.5. Thus, with k = 1, false positives and false negatives are equally penalized, with k = 2, false negatives are penalized twice as heavily as false positives, and with k = 0.5, false positives are penalized twice as heavily as false negatives.

#### Acceptable mortality

In the case study, the threshold *β*_*thresh *_was chosen so that a hospital is defined to have higher than acceptable mortality if the odds of death are 50% higher at this hospital than at an average hospital, which is consistent with analyses described by Normand et al. [16]. Therefore, *β*_*thresh *_= *β*_0 _+ log(1.5)

#### Model estimation

The following model was fit to the data:

$${\text{logit(p}}_{\text{ij}}\text{)}=\beta_{\text{0j}}+\beta_{\text{1j}}{\text{X}}_{\text{ij}}\text{, where }\left(\begin{array}{l} \beta_{0j}\\ \beta_{1j} \end{array}\right)\sim MVN\left(\left(\begin{array}{l} \beta_{0}\\ \beta_{1} \end{array}\right),\left(\begin{array}{cc} \sigma_{1}^{2} & \sigma_{12}\\ \sigma_{12} & \sigma_{2}^{2} \end{array}\right)\right).$$

The model was estimating using the Gibbs sampling implementation of Markov Chain Monte Carlo (MCMC) [29], using the BUGS software program [30]. The following proper prior distributions were assumed:

$$\left(\begin{array}{l} \beta_{0}\\ \beta_{1} \end{array}\right)\sim MVN\left(\left(\begin{array}{l} \mu_{0}\\ \mu_{1} \end{array}\right),\left(\begin{array}{cc} \tau_{11} & \tau_{12}\\ \tau_{12} & \tau_{22} \end{array}\right)\right),$$

the mean vector $\left(\begin{array}{l} \mu_{0}\\ \mu_{1} \end{array}\right)=\left(\begin{array}{r} -2.06\\ 0.91 \end{array}\right)$,

the variance-covariance matrix $\left(\begin{array}{cc} \tau_{11} & \tau_{12}\\ \tau_{12} & \tau_{22} \end{array}\right)=\left(\begin{array}{cc} 10000 & 0\\ 0 & 10000 \end{array}\right)$,

${\left(\begin{array}{cc} \sigma_{1}^{2} & \sigma_{12}\\ \sigma_{12} & \sigma_{2}^{2} \end{array}\right)}^{-1}\sim \;\text{​}Wishart\left(\left(\begin{array}{cc} 2\times 0.08139 & 2\times 0.02342\\ 2\times 0.02342 & 2\times 0.02243 \end{array}\right),2\right)$. The mean vector $\left(\begin{array}{l} \mu_{0}\\ \mu_{1} \end{array}\right)=\left(\begin{array}{r} -2.06\\ 0.91 \end{array}\right)$ was chosen based on fitting the random effects model to OMID data from 1999 (the year prior to the data used in this case study).

Twelve parallel MCMC chains were run starting from different initial values drawn from an over-dispersed distribution. Each of the 12 MCMC chains was run for an initial 5,000 burn-in iterations. Each Gibbs sampler was then monitored for an additional 10,000 iterations using a thinning interval of 10, resulting in 1,000 iterations for analysis from each sampler. Convergence of the Gibbs sampler was assessed using the Gelman-Rubin criterion for parallel runs from a Gibbs sampler [31]. The Gelman and Rubin shrink factors for the median and 97.5^th ^percentiles were all no larger than 1.01, indicating that the parallel chains had converged. All analyses were then conducted using the last 500 iterations from each of the 12 parallel chains. The 6000 sampled values (500 iterations/chain × 12 chains) from the posterior distribution were used for the subsequent analyses.

The MCMC estimate of each Bayes Rule for each of the 163 hospitals was determined using the 6000 samples from the posterior distribution of the model parameters. We then determined which hospitals were classified as having unacceptably high mortality using each of the different Bayes rules. This was done by evaluating the appropriate Bayes Rule at each iteration of the Gibbs sampler using the current sample from the posterior distribution. The posterior expectation was computed using the mean of these sampled values from the posterior distribution. If the posterior mean was greater than zero, then the hospitals was classified as having unacceptably high mortality.

## Results

The number of hospitals classified as having unacceptably high mortality ranged from a low of 1 to a high of 8, depending on which loss function was used.

When false positives and false negatives were equally penalized (k = 1 in the associated loss function), then three hospitals (hospitals A, B, and C) were classified as having unacceptably high mortality, regardless of whether 1-0 loss, absolute error loss, or squared error loss was employed.

When false positives incurred twice the penalty that false negatives incurred (k = 0.5 in the loss functions), then only one hospital (hospital A) was classified as having unacceptably high mortality when 1-0 loss and absolute error loss were employed. However, when squared error loss was employed, two hospitals were classified as having unacceptably high mortality (hospitals A and B).

When false negatives incurred twice the penalty that false positives incurred (k = 2 in the loss functions), then the number of hospitals classified as having unacceptably high mortality was either 4, 5, or 8, depending on the loss function employed. When generalized 1-0 loss was used, then eight hospitals (hospitals A, B, C, D, E, F, G, and H) were classified as having higher than acceptable mortality. When absolute error loss was used, then 5 hospitals (hospitals A, B, C, D, and E) were classified as having higher than acceptable mortality. Finally, when squared error loss was used, then four hospitals (hospitals A, B, C, and E) were classified as having unacceptably high mortality.

One hospital (hospital A) was classified as having unacceptably high mortality in all 9 analyses. In examining these results, one could conclude that hospital A would be classified as having unacceptably high mortality by all participants in the health care system.

For a given family of loss functions (generalized 1-0 loss family; generalized absolute error loss family; generalized squared error loss), the largest number of hospitals were classified as having unacceptably high mortality when false negatives were penalized more heavily than false positives (scenarios with k = 2) than when either false positives were penalized more heavily than false negatives (k = 0.5) or when false negatives and false positives were equally penalized (k = 1). Similarly, penalizing false negatives and false positives equally (k = 1) resulted in the identification of at least one additional high-mortality hospital compared to when false positives were penalized more heavily than false negatives (k = 0.5), regardless of which family the loss function came from (generalized 1-0 loss family; generalized absolute error loss family; generalized squared error loss).

**Figure 1:**
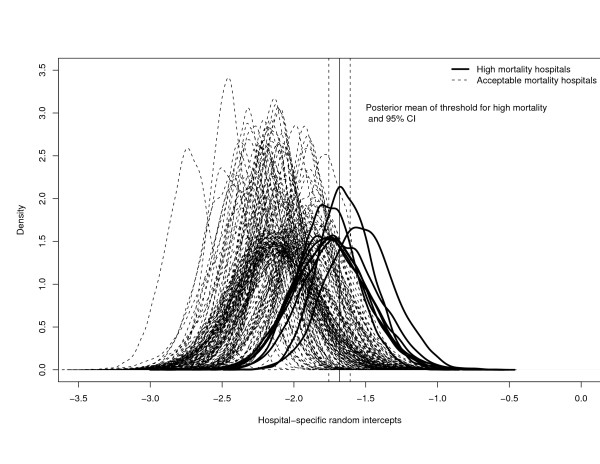
Posterior distributions of the hospital-specific random intercepts at the 163 hospitals.

**Figure 2:**
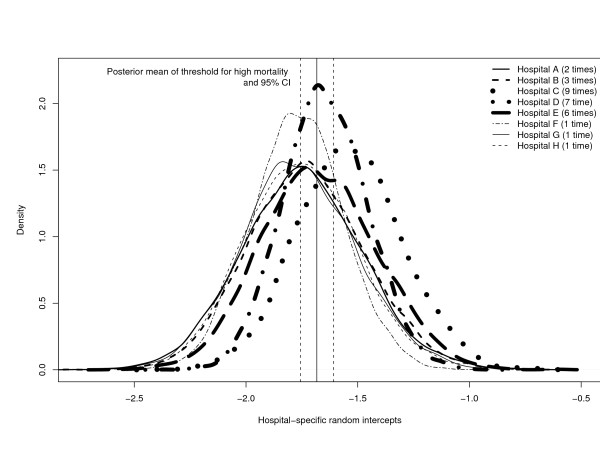
Posterior distributions of the hospital-specific random intercepts for the 8 hospitals that were classified as high-mortality outliers under at least one cost function.

In Figure 1, we depict the posterior distribution of the random intercepts for each of the 163 hospitals in the sample. The posterior distribution of the random effects for the 155 hospitals that were not classified as high-mortality outliers under any of the loss functions are depicted using dotted lines. The posterior distribution of the random effects for the 8 hospitals that were classified as high-mortality outliers under at least one loss function are depicted using heavy solid lines. We have added a solid vertical line to depict the posterior mean of the threshold for defining unacceptable high mortality (*β*_0 _+ log(1.5)), along with dashed vertical lines depicting the end points of the 95% credible interval for this threshold. In Figure 2, we display the posterior distribution of the random intercepts for the eight hospitals that were classified as high-mortality outliers using at least one loss function. One notes that of the eight hospitals that were classified as high-mortality outliers at least once, those hospitals that were identified as such more frequently tend to have posterior distributions that are shifted further to the right compared to hospitals that were identified less frequently.

For comparative purposes, model-based indirect standardization was used to identify hospitals that had higher than expected mortality [32]. This method has been used in several cardiovascular hospital report cards [2,4,7,9]. The logistic regression model adjusted for the 11 demographic and clinical variables contained in the Ontario AMI mortality prediction model that was described above. For each hospital, the ratio of observed to expected mortality (O/E ratio) was determined. Those hospitals whose O/E ratio was significantly higher than 1 were classified as having higher than expected mortality. Using this procedure, 15 out of the 169 hospitals were classified as having higher than expected mortality (hospitals A to H, in addition to seven other hospitals). Model-based indirect standardization identified substantially more hospitals as being performance outliers than did the Bayesian hierarchical method using any of the different cost functions.

## Discussion

There is a growing interest in the publication of health care report cards in which the outcomes of medical or surgical care are compared across hospitals or physicians. Several commentators have advocated for the use of Bayesian hierarchical models in provider profiling. In the current study we developed Bayes rules for identifying hospitals with higher than acceptable mortality when Bayesian hierarchical regression models are employed. We adapted three of the most commonly used loss functions (generalized 1-0 loss functions; absolute error loss; squared error loss) to the setting of provider profiling. Each of the Bayes rules that we derived was based on the posterior expectation of a function of the model parameters, the threshold for acceptable mortality, and the cost function being larger than zero. The posterior expectation of each of these functions can be calculated using MCMC methods. We illustrated our findings on a sample of 19,757 patients hospitalized with an AMI at 163 hospitals in Ontario. We found that the number of hospitals identified as having unacceptably high mortality was affected by the relative penalty assigned to false negatives compared to false positives. The choice of family of loss function tended to have less of an impact on which hospitals were classified as having higher than acceptable mortality compared to the choice of the relative penalty for false negatives compared to false positives.

The Bayes Rules for estimating parameters for generalized 1-0 loss functions, for squared error loss, and for absolute error loss are well known [24]. The latter two Bayes Rules are the mean and median of the sampled values, while the Bayes Rule for the first is the ratio of one cost of misclassification to the sum of the two costs of misclassification. While Bayes Rules are well known for estimating simple parameters, their use has not been explored in the context of using hierarchical models to identify hospitals with higher than acceptable mortality rates. In this setting, one is not estimating a parameter, but whether a parameter (the hospital-specific random effect) exceeds a specified threshold. Furthermore, the Bayes Rules that we derived for linear and quadratic loss functions are different than those for estimating simple parameters. This illustrates that prior results on Bayes Rules for simple parameters are not applicable in the setting of provider profiling.

In hospital profiling it is important to be able to correctly identify those hospitals that have unacceptably high mortality rates. For reasons of patient safety, it is important that hospitals with higher than acceptable mortality rates be correctly identified, so that the reasons for their poor performance can be investigated. However, it is also important that those hospitals that truly have acceptable mortality rates not be incorrectly identified as having higher than acceptable mortality rates. Hospitals that are incorrectly identified as having unacceptably high mortality rates face undue public criticism and damage to their reputation. Furthermore, resources are needlessly wasted in seeking to determine the reasons for the hospital's poor performance. Additionally, closing a hospital that is incorrectly identified as providing poor quality care can result in patients being denied local treatment at a hospital that truly provides acceptable quality of care. In hospital profiling there is a delicate balance that must be maintained between correctly identifying those hospitals that truly are performance outliers and not falsely labeling as outliers those hospitals that are not truly performance outliers. Different participants in health care are likely to have different perspectives on this trade-off. We have demonstrated that the choice of loss function for quantifying the cost incurred due to misclassifying hospitals can have an impact upon which hospitals are identified as having higher than acceptable mortality. There is a need for all participants in the health care system; physicians, patients, administrators, and funders; to debate the tradeoffs that have been explicitly incorporated into the analyses described in this study. Once this has been done, report cards can be explicitly produced using methodology that minimizes the cost arising from misclassification.

There is a paucity of research into developing decision-theoretic frameworks for hospital report cards. Working from a frequentist perspective and a simple model for hospital performance, Austin and Anderson derived optimal p-values required for classifying hospitals with higher than expected mortality so as to minimize the expected loss due to incorrect classification [33]. Austin and Brunner used Monte Carlo simulations to determine the accuracy of posterior tail probabilities, as measured using sensitivity and specificity, for identifying hospitals with higher than acceptable mortality [13]. In doing so, they demonstrated that the Bayes rules for generalized 1-0 loss functions were based on posterior tail probabilities. However, beyond this initial result, Bayes rules had not been developed for other, more realistic loss functions. In our case study, we found that the choice between generalized 1-0 loss, absolute error loss, and squared error loss had minimal impact upon which hospitals were identified as having unacceptably high mortality.

In conclusion, we have explicitly developed Bayes rules for several families of loss functions when Bayesian hierarchical regression models are used to identify hospitals with unacceptably high mortality. We also examined the impact of assuming different loss functions on the number of hospitals that are identified as having unacceptably high mortality.

## Conclusion

The design of hospital report cards can be placed in a decision-theoretic framework. This allows researchers to minimize costs arising from the misclassification of hospitals. The choice of loss function can affect the classification of a small number of hospitals. We found that the number of hospitals classified as having higher than acceptable mortality is affected by the relative penalty assigned to false negatives compared to false positives. However, the choice of loss function family had a lesser impact upon which hospitals were identified as having higher than acceptable mortality.

## Competing interests

The author declares that he has no competing interests.

## Pre-publication history

The pre-publication history for this paper can be accessed here:


